# Monthly Prices of Substances Used in Pharmacy

**Published:** 1814-04

**Authors:** 


### *Monthly Prices of* Substances *used in* Pharmacy.

*(To be continued regularly.)*

| | *s.* | *d.* |
|---|---|---|
| Acaciæ Gummi elect. .... lb. | 3 | 3 |
| Acidum Citricum .......... | 28 | 0 |
| ——— Benzoicum ........ | 6 | 0 |
| ——— Sulphuricum .. P. lb. | 0 | 6 |
| ——— Muriaticum ........ | 0 | 8 |
| ——— Nitricum .......... | 2 | 6 |
| ——— Aceticum .... cong. | 3 | 8 |
| Alcohol sp. gr. 815 M. lb. | 6 | 0 |
| Æther sulphuricus sp. gr. 789 | 10 | 0 |
| ——— rectificatus sp. gr. 744 | 12 | 0 |
| Ærugo ................lb. | 7 | 0 |
| Aloes spicatæ extractum .... | 5 | 6 |
| ——— vulgaris extractum .... | 4 | 4 |
| Althææ Radix ............ | 1 | 0 |
| Alumen .................. | 0 | 4 |
| Ammoniæ Murias .......... | 2 | 4 |
| ........ Carbonas ........ | 3 | 0 |
| Amygdala dulcis .......... | 5 | 8 |
| Ammoniacum (Gutt.) ...... | 6 | 6 |
| ——— (Commun.) .. | 3 | 6 |
| Anthemidis Flores opt. .... | 2 | 0 |
| Antimonium .............. | 3 | 0 |
| Antimonii Sulphuretum .... | 1 | 0 |
| Antimonium Tartarizatum .. | 5 | 0 |
| Arsenici Oxydum .......... | 0 | 10 |
| Assafœtida Gummi-resina .. | 6 | 9 |
| Aurantii Cortex .......... | 2 | 6 |
| Argenti Nitras ............ | 6 | 9 |
| Balsamum Peruvianum ...... | 33 | 0 |
| ——— Tolutanum ...... | 15 | 0 |
| Benzoinum elect. .......... | 11 | 0 |
| Calamina præparata ........ | 0 | 3 |
| Calumbæ Radix .......... | 2 | 6 |
| Cambogia opt. ............ | 9 | 6 |
| Camphora ................ | 9 | 6 |
| Canellæ Cortex .......... | 3 | 4 |
| Cardamomi Semina opt. lb. | 12 | 0 |
| Cascarillæ Cortex .......... | 6 | 6 |
| Castoreum ............nuc. | 4 | 4 |
| Catechu Extractum ........ | 2 | 3 |
| Cetaceum ................ | 3 | 0 |
| Cera alba ................ | 3 | 6 |
| ——— flava .............. | 3 | 0 |
| Cinchonæ cordifoliæ Cortex (yellow) ...... | 8 | 0 |
| ——— lancifoliæ Cortex (quilled) ...... | 13 | 0 |
| ——— oblongifoliæ Cortex (red) ........ | 16 | 0 |
| Cinnamomi Cortex ........ | 16 | 0 |
| Coccus (Coccinella) .... unc. | 4 | 0 |
| Colocynthidis Pulpæ .... M. | 8 | 0 |
| Copaiba .................. | 7 | 0 |
| Colchici Radix ............ | 3 | 7 |
| Croci stigmata ........ unc. | 4 | 0 |

| | *s.* | *d.* |
|---|---|---|
| Cupri sulphas .......... lb. | 1 | 0 |
| Cuprum ammoniatum ...... | 6 | 0 |
| Cuspariæ Cortex .......... | 4 | 0 |
| Confectio aromatica ........ | 8 | 6 |
| ——— Aurantii ........ | 2 | 9 |
| ——— Opii ............ | 4 | 6 |
| ——— Rosæ Canini ...... | 1 | 8 |
| ——— Rosæ Gallicæ .... | 2 | 0 |
| ——— Sennæ .......... | 1 | 10 |
| Emplastrum Lyttæ ........ | 6 | 6 |
| ——— Hydrargyri .... | 2 | 6 |
| Extractum Cinchonæ .. unc. | 3 | 0 |
| ——— Cinchonæ resinos. | 4 | 0 |
| ——— Colocynthidis .... | 2 | 0 |
| ——— Colocynthidis comp. | 1 | 0 |
| ——— Conii .......... | 0 | 6 |
| ——— Gentianæ ........ | 0 | 5 |
| ——— Glycyrrhizæ .. lb. | 3 | 0 |
| ——— Hæmatoxyli ...... | 0 | 5 |
| ——— Humuli ........ | 0 | 6 |
| ——— Hyoscyami .. unc. | 1 | 0 |
| ——— Jalapæ 1*s.* 6*d.* Res. | 2 | 8 |
| ——— Opii ............ | 3 | 6 |
| ——— Papaveris ........ | 0 | 6 |
| ——— Rhæi .......... | 2 | 4 |
| ——— Sarsaparillæ ...... | 0 | 10 |
| ——— Taraxaci ........ | 0 | 6 |
| Ferri carbonas .......... lb. | 0 | 9 |
| ——— sulphas ............ | 1 | 0 |
| Ferrum ammoniatum ...... | 3 | 6 |
| ——— tartarizatum ........ | 4 | 0 |
| Galbani Gum-resin. ........ | 7 | 6 |
| Gentianæ Radix .......... | 1 | 0 |
| Guaiaci Resina ............ | 6 | 0 |
| Hydrargyrus purificatus .... | 5 | 6 |
| Hydrargyri Oxymurias ...... | 8 | 0 |
| ——— Submurias ...... | 8 | 6 |
| ——— Nitrico-Oxydum .. | 8 | 6 |
| ——— Oxydum Cinereum | 16 | 0 |
| ——— Oxydum rubrum .. | 5 | 0 |
| Hydrargyrus præcipitatus albus ................ | 8 | 0 |
| Hydrargyri Sulphuretum rubrum ................ | 8 | 0 |
| Hellebori nigri Radix .... lb. | 2 | 6 |
| Ipecacuanhæ Radix ........ | 22 | 0 |
| ——— Pulvis ........ | 23 | 0 |
| Jalapæ Radix .............. | 6 | 0 |
| ——— Pulvis ............ | 6 | 6 |
| Kino .................... | 12 | 0 |
| Liquor Plumbi Acetatis M. lb. | 1 | 0 |
| ——— Ammoniæ ...... P. L | 2 | 8 |
| Linimentum Camphoræ comp. | 5 | 6 |
| Lichen Islandicus ........ lb. | 1 | 9 |
| Lyttæ .................... | 13 | 6 |

Magnesia

| | s. | d. |
|---|---|---|
| Magnesia | 8 | 6 |
| ——— Carbonas | 2 | 6 |
| ——— Sulphas | 1 | 4 |
| Manna optima | 8 | 6 |
| ——— communis | | |
| Moschus pod, 24s. in gr. unc. | 40 | 0 |
| Mastiche lb. | 5 | 6 |
| Myristicæ Nuclei | 25 | 0 |
| Myrrha elect. | 6 | 6 |
| Olibanum | 3 | 0 |
| Opoponax | 23 | 0 |
| Opium (Turkey) | 34 | 0 |
| ——— (East India) | | |
| Oleum Amygdale lb. | 5 | 4 |
| ——— Anisi unc. | 2 | 6 |
| ——— Anthemidis | 5 | 0 |
| ——— Cinnamomi Cassia | 8 | 0 |
| ——— Caryophilli | 4 | 6 |
| ——— Cajuputi | 8 | 0 |
| ——— Carui | 1 | 3 |
| ——— Juniperi | 0 | 9 |
| ——— Lavandulæ | 4 | 0 |
| ——— Lini cong. | 8 | 6 |
| ——— Menthæ piperitæ ung. | 4 | 6 |
| ——— viridis | 3 | 0 |
| ——— Olivæ cong. | 35 | 0 |
| ——— secundum | | |
| ——— Pimentæ unc. | 4 | 6 |
| ——— Ricini optim. (per bottle) | 10 | 0 |
| ——— Rosmarini unc. | 0 | 9 |
| ——— Succini 2s. 4d. rect. | | |
| ——— Sulphuratum M. lb. | 1 | 6 |
| ——— Terebinthinæ | 2 | 0 |
| ——— rectificatum | | |
| Papaveris Capsulæ (per 100) | 2 | 4 |
| Plumbi Carbonas lb. | 0 | 8 |
| ——— Superacetas | 2 | 0 |
| ——— Oxydum semi-vitreum | 0 | 6 |
| Potassa Fusa unc. | 0 | 6 |
| ——— cum Calce | | |
| Potassæ Nitras lb. | 1 | 0 |
| ——— Acetas | 8 | 0 |
| ——— Carbonas | 1 | 0 |
| ——— Supercarbonas | 4 | 0 |
| ——— Sulphas | 1 | 0 |
| ——— Sulphuretum unc. | 0 | 2 |
| ——— Supersulphas | 0 | 4 |
| ——— Tartras lb. | 3 | 0 |
| ——— Supertartras | 2 | 0 |
| Pilulæ Hydrargyri unc. | 0 | 6 |
| Pulvis Antimonialis | 0 | 6 |
| Resina Flava lb. | 0 | 5 |
| Rhæi Radix (Russia) | 30 | 0 |
| ——— (East India) | 12 | 0 |
| Rosæ Folia | 10 | 6 |
| Sapo (Spanish) | 2 | 6 |
| Sarsaparilla Radix | 5 | 6 |
| Scammoneæ Gum-Resina unc. | 2 | 3 |
| Scillæ Radix siccat lb. | 4 | 6 |
| Senegæ Radix | | |
| Sennæ Folia | 6 | 0 |
| Serpentariæ Radix | | |
| Simaroubæ Cortex | 4 | 0 |
| Sodæ Boras | 3 | 9 |
| ——— Sulphas | 0 | 6 |
| ——— Carbonas | 7 | 0 |
| ——— Subcarbonas | 1 | 4 |
| ——— exsiccata | 2 | 8 |
| Soda tartarizata | 2 | 4 |
| Spongia usta | 20 | 0 |
| Spiritus Ammoniæ M. lb. | 4 | 0 |
| ——— aromaticus | 4 | 6 |
| ——— fœtidus | 3 | 6 |
| ——— succinatus | 4 | 6 |
| ——— Cinnamomi | 3 | 6 |
| ——— Lavandulæ | 4 | 6 |
| ——— Myristicæ | 3 | 0 |
| ——— Pimentæ | 3 | 0 |
| ——— Rosmarini | 4 | 0 |
| ——— Ætheris Aromaticus | 6 | 0 |
| ——— Compositus | 6 | 0 |
| ——— Nitrici sp. gr. 860 | 5 | 6 |
| ——— Sulphurici | 6 | 0 |
| ——— Vin rectificatus cong. | 28 | 0 |
| Syrupus Papaveris lb. | 2 | 0 |
| Sulphur | | |
| ——— Sublimatum | 0 | 6 |
| ——— Lotum | 1 | 0 |
| ——— Præcipitatum | 1 | 0 |
| Tamarindus opt. | 2 | 6 |
| Terebinthina Vulgaris | 0 | 9 |
| ——— Canadensis | | |
| ——— Chia | | |
| Tragacantha Gummi | 10 | 0 |
| Valerianæ Radix | 1 | 8 |
| Veratri Radix | 1 | 6 |
| Unguentum Hydrargyri-fortius | 5 | 0 |
| ——— Nitratis | 3 | 3 |
| ——— Nitrico-oxy. | 2 | 9 |
| Uvæ Ursi Folia | 2 | 4 |
| Zinci Oxydum | 4 | 6 |
| ——— Sulphas purif. | 2 | 0 |
| Zingiberis Radix opt. | 2 | 4 |

English Leeches 14s. per dozen.

Foreign Leeches, in general as good as the English, 36s. to 40s. per hundred.

*Price of Vials per Gross.*—8 oz. 70s.—6 oz. 58s.—4 oz. 47s.—3 oz. 43s.—2 oz. 36s.—1 oz. 30s.—½ oz. 24s.

